# Regional Brain [^11^C]carfentanil Binding Following Tobacco Smoking

**DOI:** 10.1016/j.pnpbp.2015.01.007

**Published:** 2015-01-15

**Authors:** Edward F Domino, Mika Hirasawa-Fujita, Lisong Ni, Sally K Guthrie, Jon Kar Zubieta

**Affiliations:** 1 Department of Pharmacology, University of Michigan, Ann Arbor, MI USA; 2 College of Pharmacy, University of Michigan, Ann Arbor, MI USA; 3 Department of Psychiatry, University of Michigan, Ann Arbor, MI USA

**Keywords:** PET, [^11^C]carfentanil, OPRM1, A118G, smoking

## Abstract

**Objective:**

To determine if overnight tobacco abstinent carriers of the AG or GG (*G) vs. the AA variant of the human mu opioid receptor (OPRM1) A118G polymorphism (rs1799971) differ in [^11^C]carfentanil binding after tobacco smoking.

**Methods:**

Twenty healthy American male smokers who abstained from tobacco overnight were genotyped and completed positron emission tomography (PET) scans with the mu opioid receptor agonist, [^11^C]carfentanil. They smoked deniconized (denic) and average nicotine (avnic) cigarettes during the PET scans.

**Results:**

Smoking avnic cigarette decreased the binding potential (BP_ND_) of [^11^C]carfentanil in the right medial prefrontal cortex (mPfc; 6,56,18), left anterior medial prefrontal cortex (amPfc; −2,46,44), right ventral striatum (vStr; 16, 3, −10), left insula (Ins; −42,10, −12), right hippocampus (Hippo; 18, −6, −14) and left cerebellum (Cbl; −10, −88, −34), and increased the BP_ND_ in left amygdala (Amy; −20,0, −22), left putamen (Put; −22, 10, −6) and left nucleus accumbens (NAcc; −10,12, −8). In the AA allele carriers, avnic cigarette smoking significantly changed the BP_ND_ compared to after denic smoking in most brain areas listed above. However in the *G carriers the significant BP_ND_ changes were confirmed in only amPfc and vStr. Free mu opioid receptor availability was significantly less in the *G than the AA carriers in the Amy and NAcc.

**Conclusion:**

The present study demonstrates BP_ND_ changes induced by avnic smoking in OPRM1 *G carriers were blunted compared to the AA carriers. Also *G smokers had less free mu opioid receptor availability in Amy and NAcc.

## 1. Introduction

Many studies with mice have demonstrated that nicotine induces endogenous brain opioid release (Davenport et al., 1990; Dhatt et al., 1995; Isola et al., 2009). Furthermore C57BL/6 mice treated with large doses of nicotine results in marked tolerance to nicotine antinociception (Galeote et al., 2006). The C57B4/6 mu opioid knockout mice also develop tolerance to nicotine antinociception more quickly. The antinociceptive actions of nicotine in rodents are not reduced by mu opioid antagonists. In humans, nicotine/tobacco smoking is not an effective analgesic. However, some brain evoked potentials due to painful laser stimuli are reduced, but C fiber effects are enhanced by tobacco smoking (Miyazaki et al., 2009; Miyazaki et al., 2010).

Additional basic science studies support the importance of the opioid system, especially the mu opioid receptor (OPRM1), in drug addiction. Humanized h/mOPRM1-118 AA or h/mOPRM1-118 GG receptors (knockin) mice show different reinforcement of alcohol. The GG mice have a four-fold greater vStr/NAcc DA release to alcohol than the former (Ramchandani et al., 2011). Also this gene is involved in opiate and cocaine addiction and treatment (Kreek et al., 2005). Recently Zhang et al. (2015) found that GG mice self-administrated more heroin and had more brain dopamine release in response to heroin than AA mice. In mice with a lack (knockout) of the mu opioid receptor, ethanol and cocaine (Becker et al., 2002) and nicotine (Berrendero et al., 2002; Walters et al., 2005) are not rewarding. Furthermore mice with the G allele of A112G SNP (which is equivalent to human OPRM1 A118G SNP) have reduced receptor protein, less morphine induced hyperactivity, and less locomotor sensitization. Additionally, Female mice have less morphine reward aversive naloxone precipitated withdrawal (Mague et al., 2009).

Ray et al. (2011) reported that smokers with the OPRM1 *G allele have reduced [^11^C]carfentanil binding potentials (MOR BP_ND_) compared to AA carriers with 0.6 mg of nicotine (nic) cigarette smoking. Reduced BP_ND_ assumes increased endogenous opioid release and less free mu opioid receptors (activation). They also found that *G carriers had a positive association between decreased MOR BP_ND_ and smoking reward. The present study reports the role of OPRM1 A118G in brain endogenous opioid release following tobacco smoking as measured by [^11^C]carfentanil displacement with denic and 1.0 mg nicotine avnic cigarettes.

## 2. Materials and Methods

Twenty four healthy American males were recruited for this study. Four of 24 subjects were omitted due to incomplete PET scans, blood samples, and greater than 10 ng/mL boost of plasma nicotine levels after smoking. In this study the subjects were all smokers who smoked 15–40 cigarettes per day for at least one year. These are the same subjects who participated in the published PET study with [^11^C]raclopride by (Domino et al., 2012). The two counter balanced PET scan with both [^11^C]raclopride and [^11^C]carfentanil were done on two separate days. Each session was designed to have the volunteers inhale tobacco smoke from either two denic or avnic cigarettes with either [^11^C]raclopride or [^11^C]carfentanil. Detailed subject demographics, experimental design, PET scanning protocol, image and data acquisition, data analysis and genotyping were described in the previous published study (Domino et al., 2012). However for the [^11^C]carfentanil SPM5 ROI analysis, the threshold p< 0.01 and the extent threshold K=10 voxels were used in this study. It is important to note that due to the University of Michigan Hospital No Smoking rule the smoke of two denic or avnic cigarettes was inhaled from an enclosed gallon bottle. The smoke was exhaled into a vacuum purging system and released into the environment on the roof of the hospital. The present study describes the results with [^11^C]carfentanil with a total mean ± S.E. mass of carfentanil of 16.8 pg/kg per scan.

### 2.1 Statistical analyses

The OPRM1 A118G SNP effects on BP_ND_ were analyzed with using EZR version 1.2121 (Kanda, 2013). The p value was considered as significant if p < 0.05. In all figures significance are indicated as *p< .05, **p< .01 and *** p< .001. Effect size, Power, and sample size for Power = 0.8 with actual Effect size were calculated with G*Power version 3.1.9.222 (Faul et al., 2007). The data were analyzed with a post-hoc analysis, and the significance level α was fixed at 0.05 for all analyses.

## 3. Results

### 3.1 Tobacco smoking effects on [^11^C]carfentanil BP_ND_

**Figure 1:**
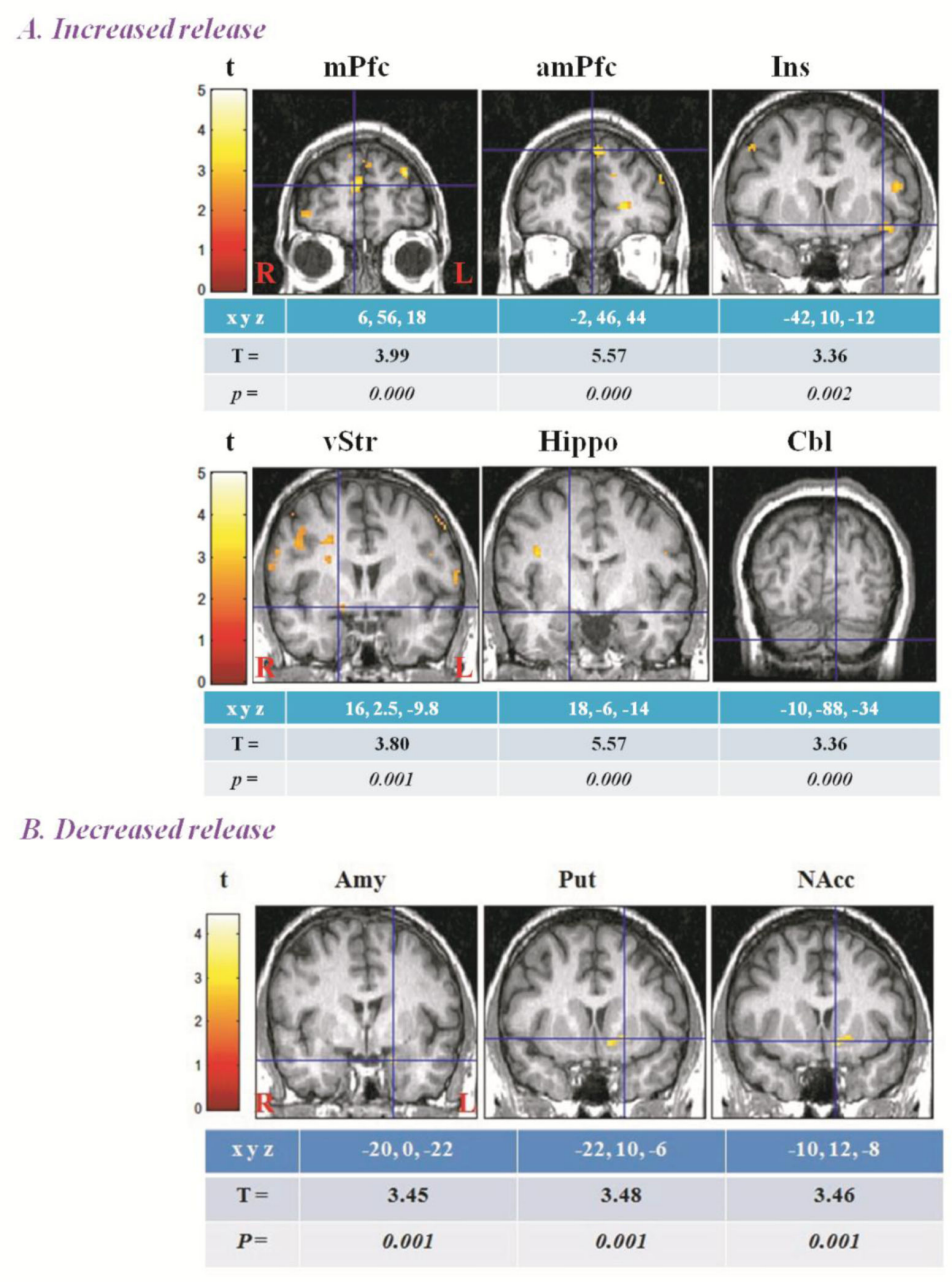
A: Increased and B: Decreased endogenous mu opioid release (N=20)

First all 20 male subjects were compared as one group. Tobacco smoking altered regional brain endogenous mu opioid release. The decreased [^11^C]carfentanil BP_ND_ after smoking denic cigarettes minus the BP_ND_ after smoking avnic cigarettes was found in six regional brain areas. The decrease in the BP_ND_ was interpreted as increases in endogenous mu opioid release (activation). Significant decreases in BP_ND_ after avnic compared to after denic were found in the ventral striatum (vStr), left insula (Ins), right hippocampus (Hippo), left anterior medial prefrontal cortex (amPfc), left medial prefrontal cortex (mPfc), and left cerebellar vermis (Cbl; Figure 1A). The BP_ND_s after denic and avnic smoking were 0.81±0.68, 0.67±0.60, 2.08±0.31, 1.95±0.27, 1.91±0.68, 1.76±0.60, 1.75±0.44, 1.61±0.40, 1.95±0.39, 1.86±0.38, 1.15±0.52 and 1.01±0.53 in the brain areas listed above.

In contrast increased [^11^C]carfentanil BP_ND_ after smoking avnic cigarettes minus the BP_ND_ after smoking denic cigarettes occurred in three regional brain areas. The significant increase in the BP_ND_ after smoking was interpreted as a decrease in endogenous opioid release (deactivation) in the amygdala (Amy), nucleus accumbens (NAcc), and putamen (Put) in the left hemisphere (Figure 1B). The BP_ND_s after denic and avnic smoking were 1.33±0.69, 1.12±0.59, 1.40±0.66, 1.23±0.61, 1.32±0.51 and 1.16±0.52 in the above brain areas. The values of [^11^C]carfentanil BP_ND_ are similar to those of (Ray et al., 2011).

### 3.2 OPRM1 genotype comparisons on tobacco smoking effects and [^11^C]carfentanil BP_ND_

The subjects were genotyped and separated into two groups based on the existence OPRM1 G allele. There were 15 AA and 5 *G (GA and GG) carriers. For each genotype, BP_ND_s after denic and avnic smoking were compared in various regional brain areas. In AA allele carriers, BP_ND_s were significantly different between after denic and after avnic smoking: vStr (t=2.75, df=14, p=0.02), left Ins (t=2.91, df=14, p=0.01), right Hippo (2.83, df=14, p=0.01), left amPfc (3.92, df=14, p=0.002), left mPfc (4.35, df=14, p<0.001), left Cbl (t=3.97, df=14, p=0.001), Amy (t=4.39, df=14, p<0.001) and NAcc (t=2.73, df=14, p=0.02). In the *G carriers, the BP_ND_s were significantly different between cigarette smoking conditions in vStr (t=3.57, df=4, p=0.02) and left amPfc (t=2.95, df=4, p=0.04).

### 3.3 OPRM1 A118G genotype differences on free mu opioid receptor availability

**Figure 2:**
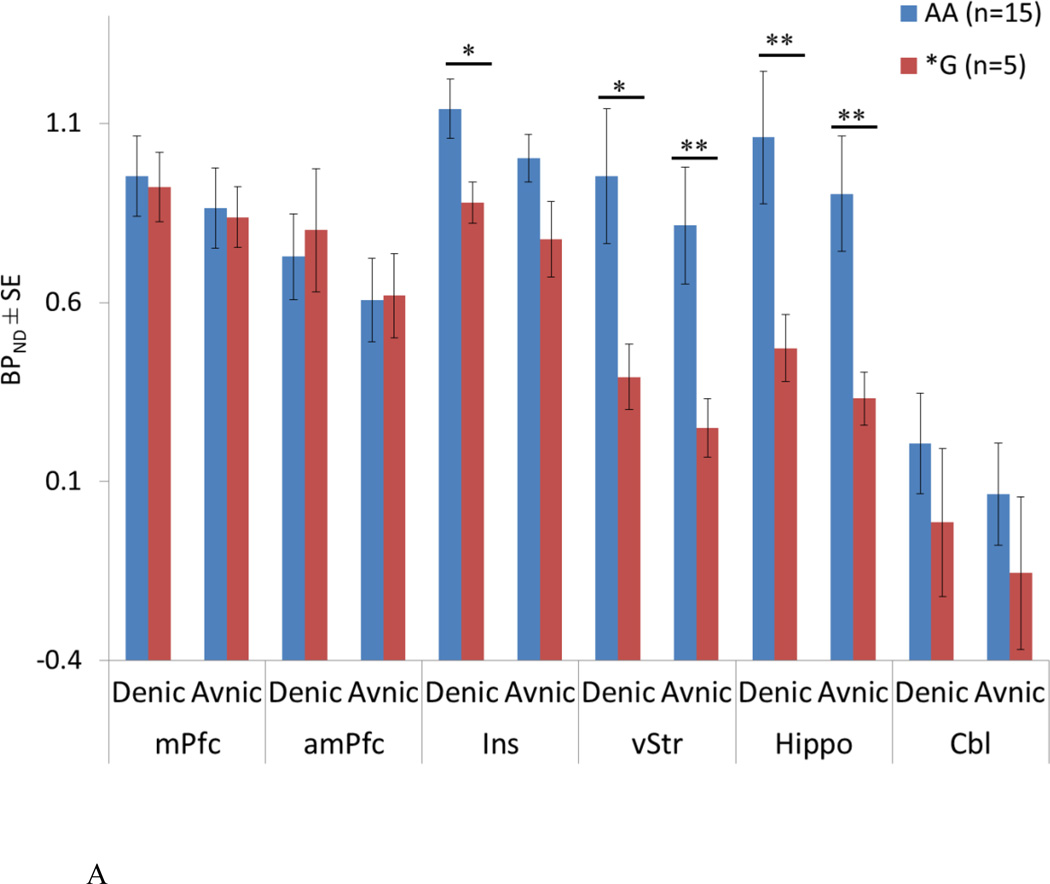

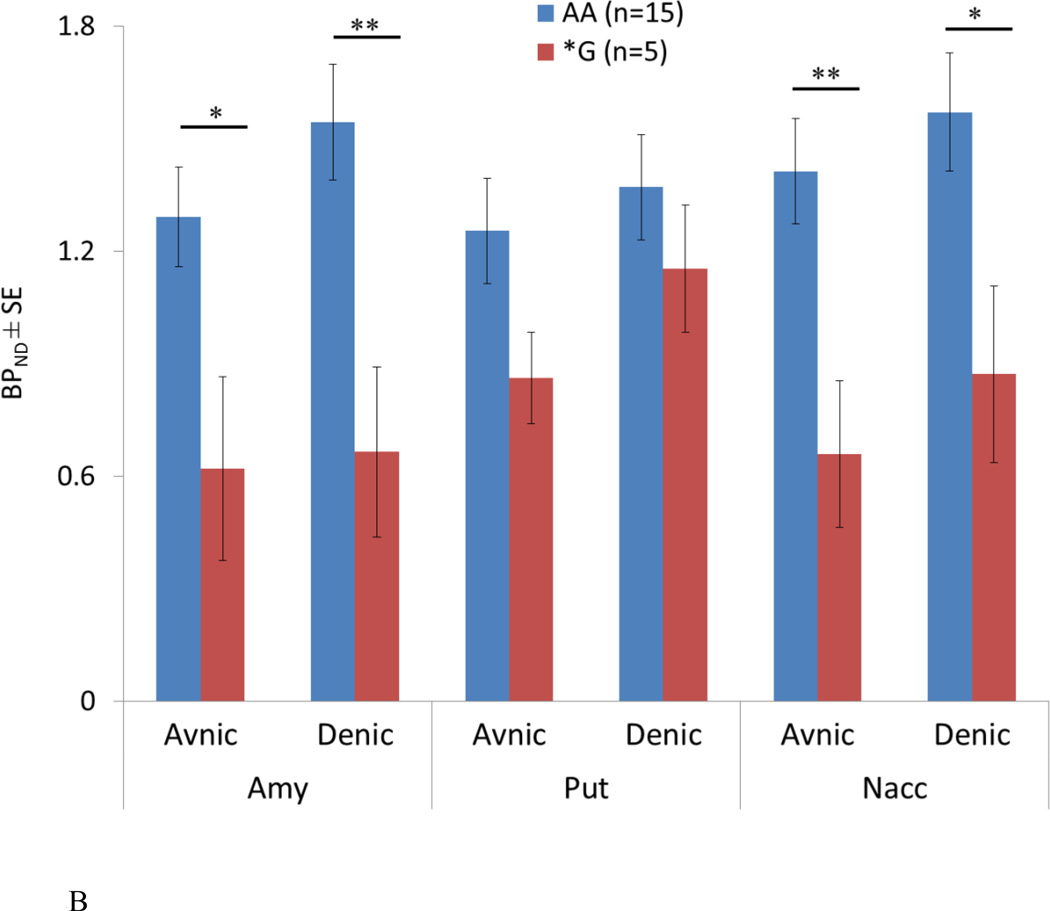
A.OPRM1 A118G effects on increases in endogenous mu opioid release B.OPRM1 A118G effects on decreases in endogenous mu opioid release

There were fewer free mu opioid receptors in the left Ins (denic: t=2.58, df=16.9, p=0.02, surprisingly avnic effects were not significant), vStr (denic: t=2.67, df=17.9, p=0.02. avnic: t=3.10, df=17.9, p=0.006) and right Hippo (denic: t=2.83, df=18.0, p=0.01. avnic: t=3.22, df=17.8, p=0.005; Figure 2A) left Amy (avnic: t=2.40, df=6.53, p=0.05. denic: t=3.20, df=8.06, p=0.01) and NAcc (avnic: t=3.14, df=8.51, p=0.01. denic: t=2.47, df=7.98, p=0.04; Figure 2B) in the *G compared to AA carriers.

### 3.4 Effect size and Power analyses

The results of the Power analyses for denic and avnic cigarette smoking effects on BP_ND_ changes within the OPRM1 variant are summarized in Table 1A (for activation) and B (for deactivation). For the AA carriers Effect size and Power were adequate to conclude that avnic cigarette smoking changes BP_ND_ significantly. However for the *G carriers Effect size and Power were relatively small to determine smoking effects.

Although the adequate p values for left Ins and right Hippo for comparison between the variants as illustrated in the Results section, Power analyses revealed poor statistical Power for OPRM1 genotype differences in endogenous mu opioid release in these brain areas (Table 2A).

In contrast Power analyses for OPRM1 genotype differences on decreases in endogenous mu opioid release indicated adequate Effect size and Power in left Amy (after denic) and NAcc (after avnic; Table 2B).

## 4. Discussion

Zhang et al. (2005) reported that mRNA and receptor protein expression levels are lower in the G receptor variant. Subsequently, several studies in mice also indicate that the A118G variant has less receptor protein expression (Mague et al., 2009; Wang et al., 2012). There are five asparagine (N) residues as putative N-glycosylation sites on the N terminus of OPRM1 (Comprehensive Amino Acids Sequences Annotated database; CAAS db). N-glycosylation is considered as an important process for stabilization and biosynthesis of glycoproteins (Solá and Griebenow, 2009). The receptor with 118G allele has smaller molecular mass and less stability compare to wild type mice due to the lack of N-glycosylation (Peng et al., 2012). However the G variant has greater affinity for the endogenous opioid β-endorphin compared to A118A variant in AV-12 fused cells (Bond et al., 1998) but no change in binding affinity in HEK293 cells (Beyer et al., 2004).

In humans, Ray et al. (2006) used the number of cigarette puffs as a measure of nicotine reinforcement. They found that *G allele women smokers took fewer puffs than males smoking after two hours of tobacco abstinence. The women did not distinguish between smoking denic or low nicotine (0.6 mg) cigarettes. Especially important, after 50 mg of oral naltrexone, but not after placebo, all of the *G smokers had lower difference liking scores than did AA smokers. Lerman et al. (2004) reported that the G allele carriers were more likely to quit smoking successfully using nicotine replacement therapy. Furthermore Zhang et al. (2006) found in 688 Caucasian smokers and nonsmokers that three OPRM1 SNPs (rs9479757, rs2075572, and rs10485057) were highly significant for tobacco smoking initiation (p = 0.0002), and marginal for nicotine dependence (p = 0.05). Recently Verhagen et al. (2012) published a meta-analysis about smoking initiation, nicotine dependence and smoking cessation. They concluded that subjects with the OPRM1 AA allele had higher risk for nicotine dependence. These results indicated evidence of the association between the mu opioid system and tobacco use.

In the present study, both increases and decreases in endogenous mu opioid release following tobacco smoking were observed. In some agreement with our preliminary study by Scott et al. (2007) increased release was observed in the right mPfc and amPfc and decreased release in left Amy, Put, and NAcc. The vStr is activated with smoking related cues (David et al., 2005). Sustained damage to the Ins interrupt tobacco smoking (Naqvi et al., 2007). The Hippo is related to tobacco smoking via nicotinic acetylcholine receptors which induce endogenous opioid release. Chronic nicotine exposure increases receptor expression levels in the brain which results in a positive correlation between degree of tobacco use and the number of receptor binding sites (Breese et al., 1997). The Pfc is strongly activated with nicotine (Schroeder et al., 2001). Nicotine increases the rates of local cerebral glucose utilization which indicates nicotine binding receptors in several brain regions including Cbl (London et al., 1988). Koob and Le Moal (2001) reported Amy and opioid peptide neurons within this site are major components of a drug reward circuit. Galeote et al. (2006) reported repeated nicotine administrations decreases the density of mu opioid receptors in the caudate-Put as well as in the core and shell of NAcc in mice.

The present study is only in some agreement with Ray et al. (2011). They reported that OPRM1 AA tobacco smokers had more free brain mu opioid receptors than *G carriers. In this study in the Ins, vStr, Hippo, Amy and NAcc, the *G allele carriers also had less free mu opioid receptor density compared to the AA carriers. However our power analyses indicated that Effect size and Power were adequate only for amygdala and Nacc. As mentioned above in the Results section, smoking effects were confirmed in the AA carriers with increases and decreases in free mu opioid receptor levels between denic and avnic cigarette smoking. However the BP_ND_ of *G carriers after avnic smoking were only significantly different in vStr and amPfc compared to that of after denic cigarette smoking. This suggests that tobacco smoking/nicotine effects on endogenous mu opioid release were blunted in the *G carriers supporting the concept of a “loss-of function” receptor (Mague et al., 2009). Effect size and Power were acceptable for the AA carriers, but a larger number of *G allele carrier is needed.

The present study has major limitations: (1) Limited number of subjects. (2) Lack of controls for the tobacco smokers. (3) Females were not studied (due to a lack of approved research funds for the role of sex differences). The number of G allele carriers was also limited. Larger groups including tobacco smokers and nonsmokers, males as well as females must be studied for Effect size and Power to be >0.8. (4) Since [^11^C]carfentanil is potent selective mu opioid receptor agonist, its binding affinity may differ between SNP variants. Identifying the cell types releasing various types of endogenous opioids were not examined. The mass dose used in this study is in a common range (20–30 pg/kg) for human PET studies with [^11^C]carfentanil (Colasanti et al., 2012; Ray et al., 2011; Wager et al., 2007; Wand et al., 2011). The dose of [^11^C]carfentanil is less than 15 % of the therapeutic dose for conscious sedation (Cortinez et al., 2005; Goodman et al., 2006) and is only effective as a radiotracer but not as a agonist to release endogenous opioids. One may conclude that specific MOR BP_ND_ was only measured in this study. The BP_ND_ after denic smoking was considered the baseline to compensate for non-specific binding (which was unlikely to occur). Before tobacco smoking MOR BP_ND_ in abstinent smokers and nonsmokers should be studied.

In conclusion this small genetic/PET study indicates that the OPRM1 A118G genotype altered tobacco smoking/nicotine effects on endogenous brain opioid release. The AA allele smokers had more free mu opioid receptor availability.

## Figures and Tables

**Table 1:** 

A. Power analyses for denic and avnic smoking effects on activation of endogenous mu opioid releases within the variant												
	mPfc		amPfc		Ins		vStr		Hippo		Cbl	

xyz coordinates	−6,56,18		2,46,44		42, 10, −12		16, 2.5, −9.8		−18, −1, −8		10,−88,−34	

	AA	*G	AA	*G	AA	*G	AA	*G	AA	*G	AA	*G

Effect size	1.12	0.46	1.02	1.32	0.75	0.61	0.71	1.60	0.73	1.22	1.03	1.12

Power	0.98	0.12	0.94	0.57	0.75	0.18	0.70	0.73	0.73	0.51	0.95	0.45

B. Power analyses for denic and avnic smoking effects on deactivation of endogenous mu opioid releases within the variant						
	Amy		Put		NAcc	

xyz coordinates	20, −1, −22		22,10,−6		10,12,−8	

	AA	*G	AA	*G	AA	*G

Effect size	1.13	0.15	0.42	1.53	0.64	0.85

Power	0.98	0.06	0.32	0.69	0.61	0.29

**Table 2:** 

A. Power analyses for OPRM1 A118G differences on increases in endogenous mu opioid release												
	mPfc		amPfc		Ins		vStr		Hippo		Cbl	
xyz coordinates	−6,56,18		2,46,44		42, 10, −12		16, 2.5, −9.8		−18, −1, −8		10,−88,−34	
Cigarettes smoked	Denic	Avnic	Denic	Avnic	Denic	Avnic	Denic	Avnic	Denic	Avnic	Denic	Avnic
Effect size	0.08	0.05	0.16	0.02	0.93	0.83	1.04	1.22	0.9	1.03	0.42	0.44
Power	0.05	0.05	0.06	0.05	0.38	0.32	0.46	0.58	0.36	0.45	0.12	0.12

B. Power analyses for OPRM1 A118G differences on decreases in endogenous mu opioid release						
	Amy		Put		NAcc	
xyz coordinates	20, −1, −22		22,10,−6		10,12,−8	
Cigarettes smoked	Avnic	Denic	Avnic	Denic	Avnic	Denic
Effect size	1.26	1.59	0.91	0.46	1.53	1.23
Power	0.61	0.81	0.37	0.13	0.78	0.59

## Highlights

- Authors found that average nicotine (avnic) cigarette smoking decreases [^11^C]carfentanil binding potentials (BP_ND_) in several regional brain areas among overnight abstinent male tobacco smokers.
- In the OPRM1 AA allele carriers, avnic cigarettes significantly changed BP_ND_ compared to after denicotinized (denic) smoking in the several regional brain regions.
- For the OPRM1 *G carriers the significant BP_ND_ changes were confirmed in only limited brain areas compared to the AA carriers.
- The *G allele carriers had fewer free brain mu opioid receptor availability than the AA carriers in left amygdala and left nucleus accumbens.
